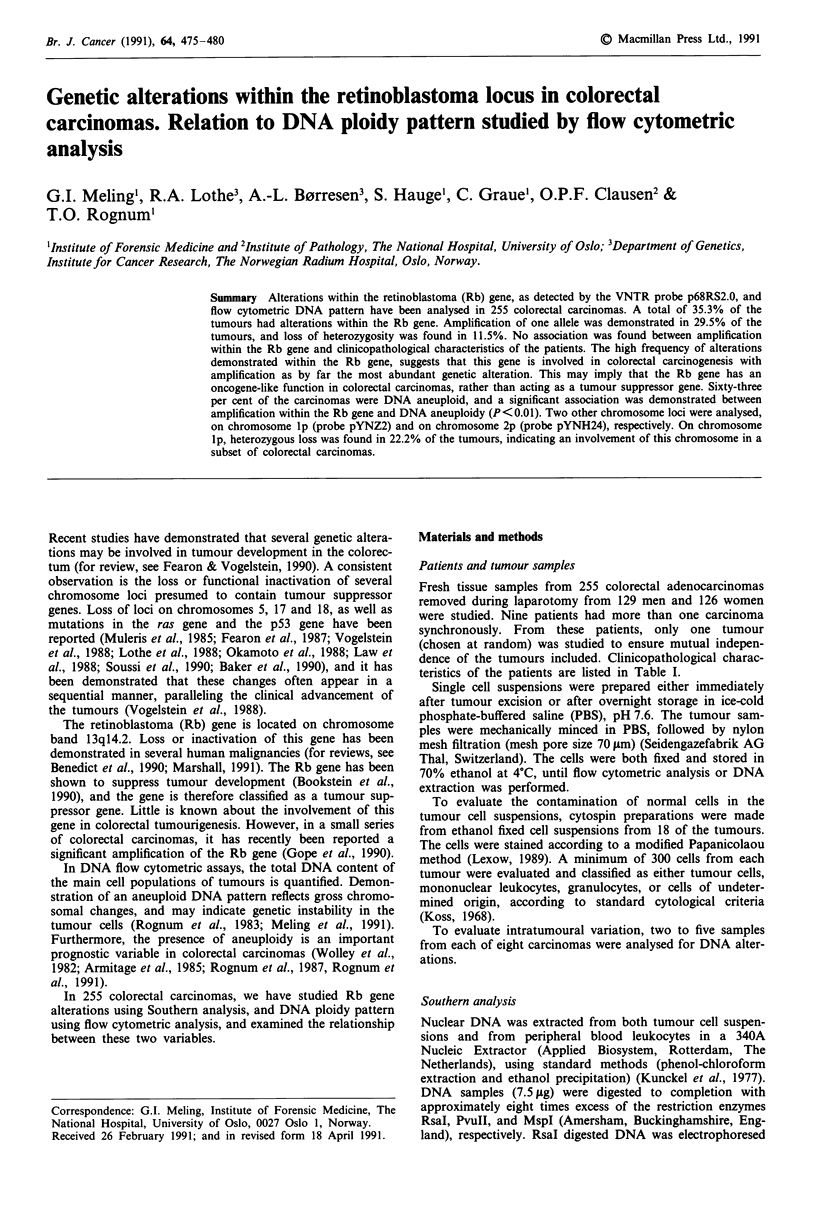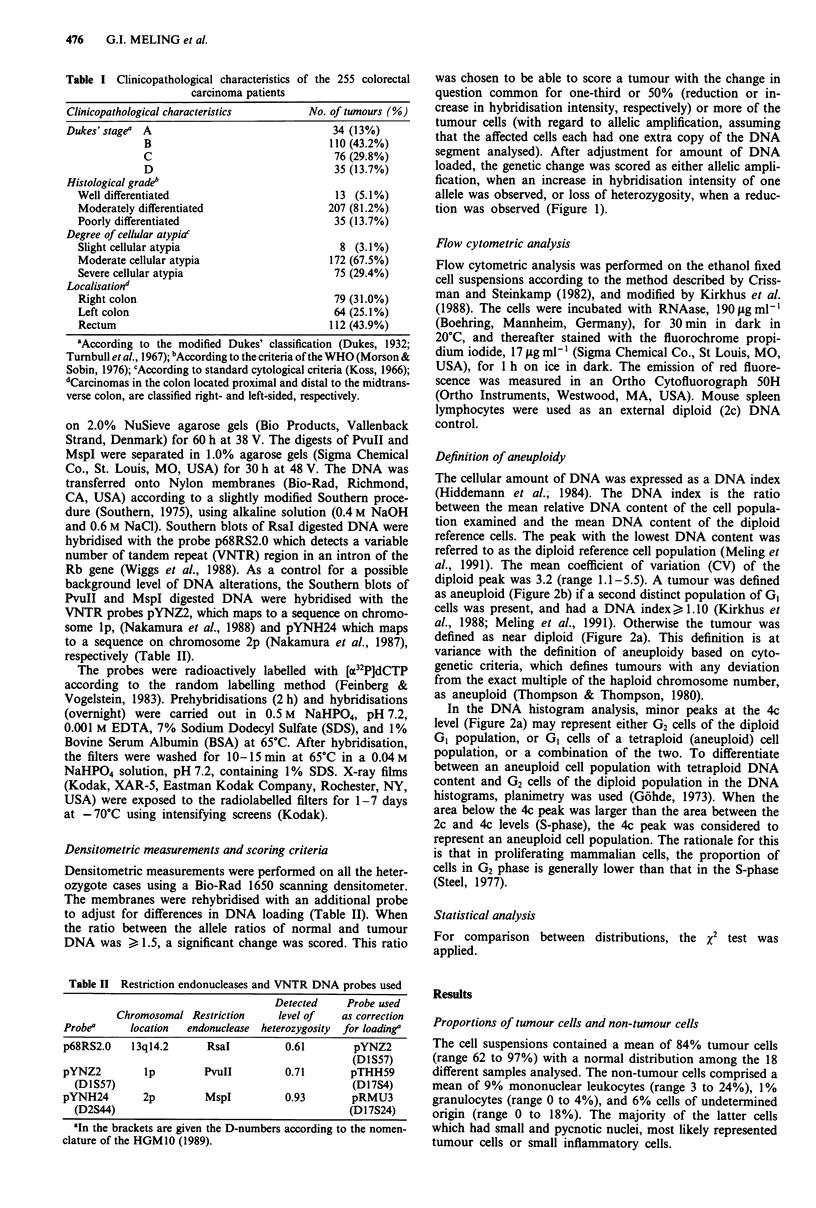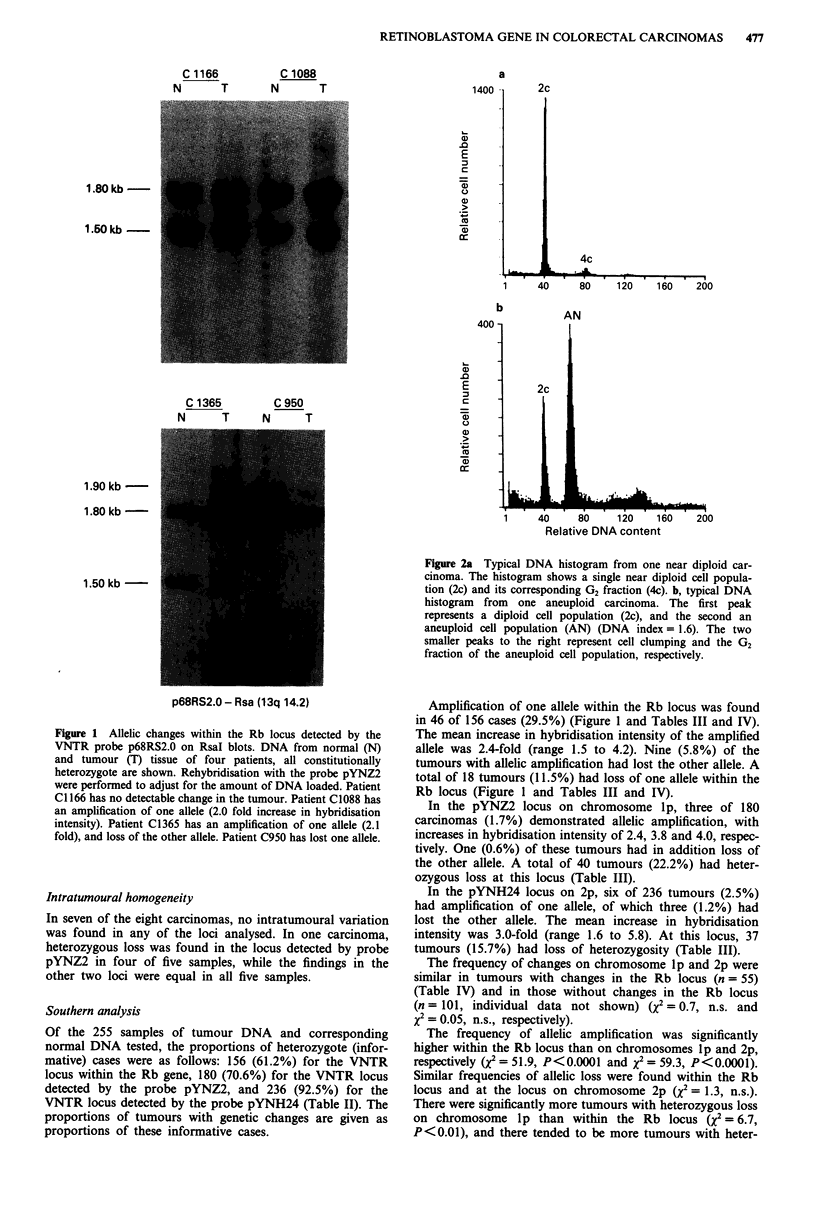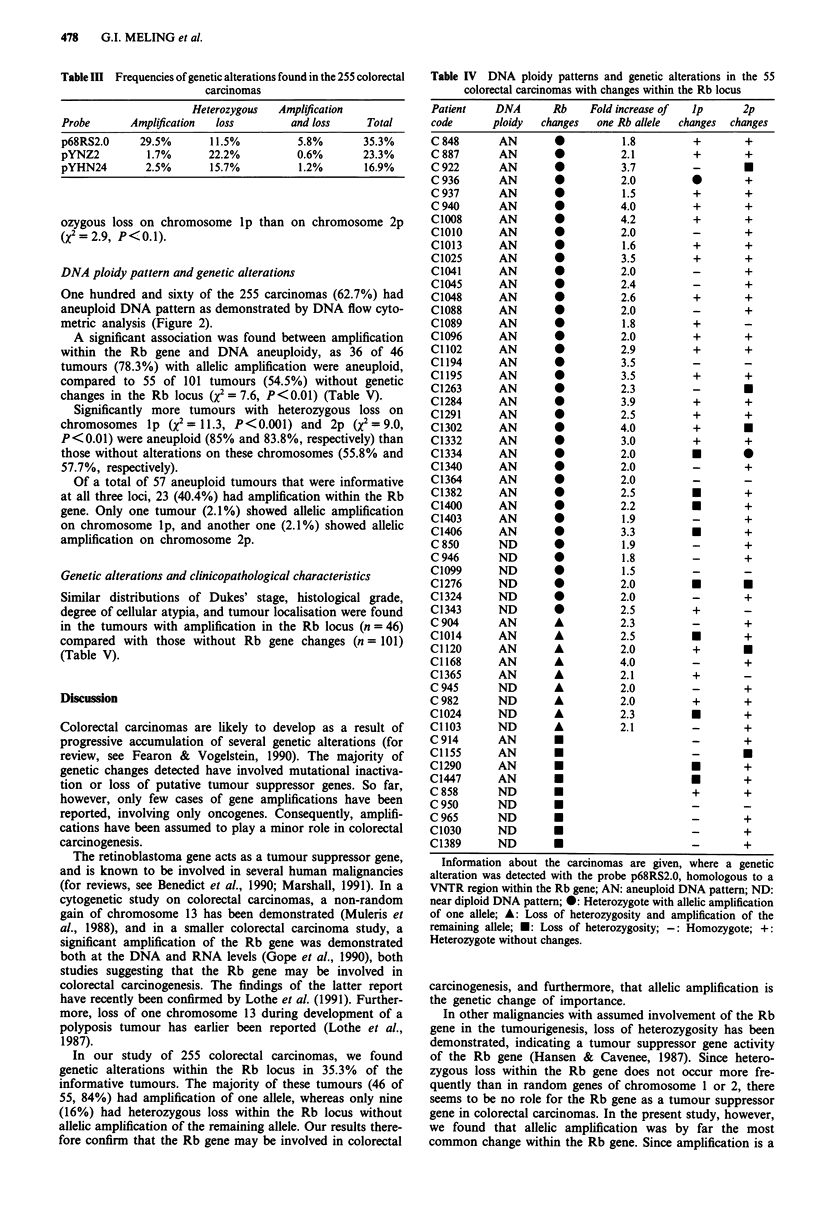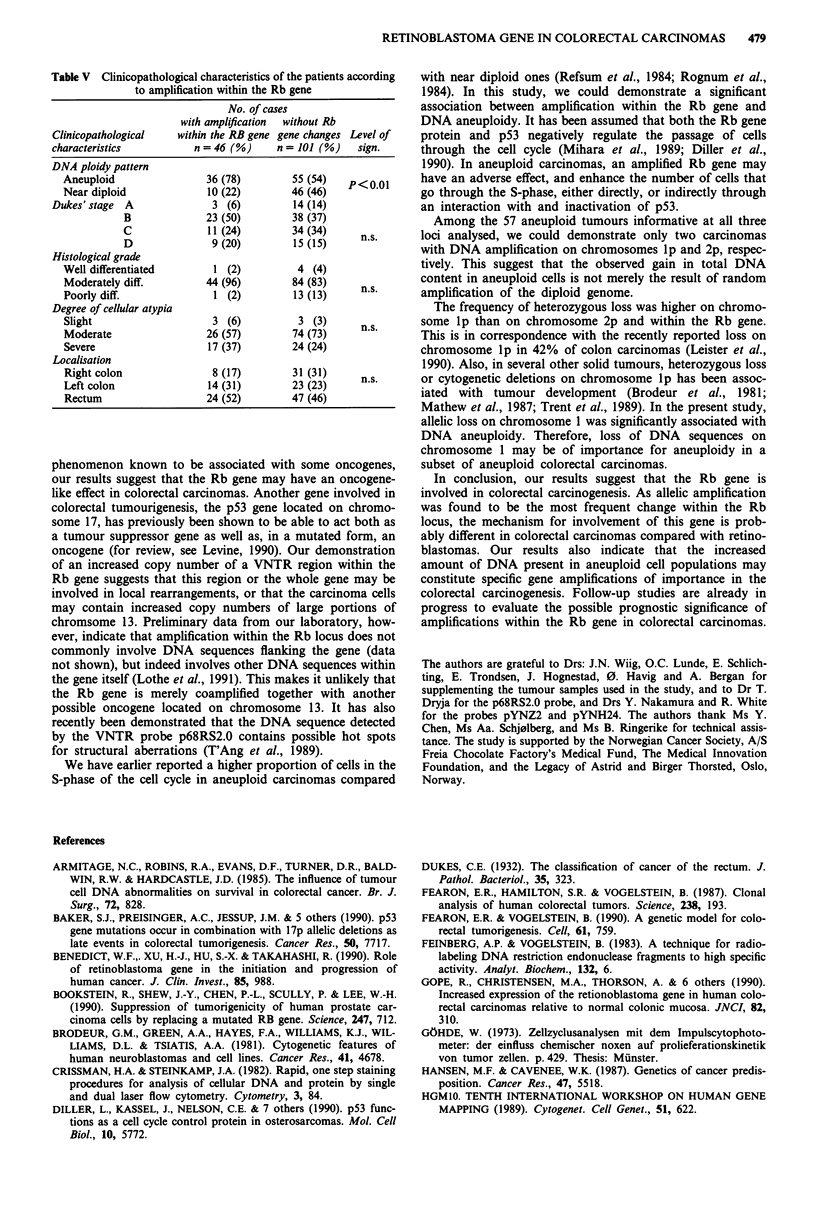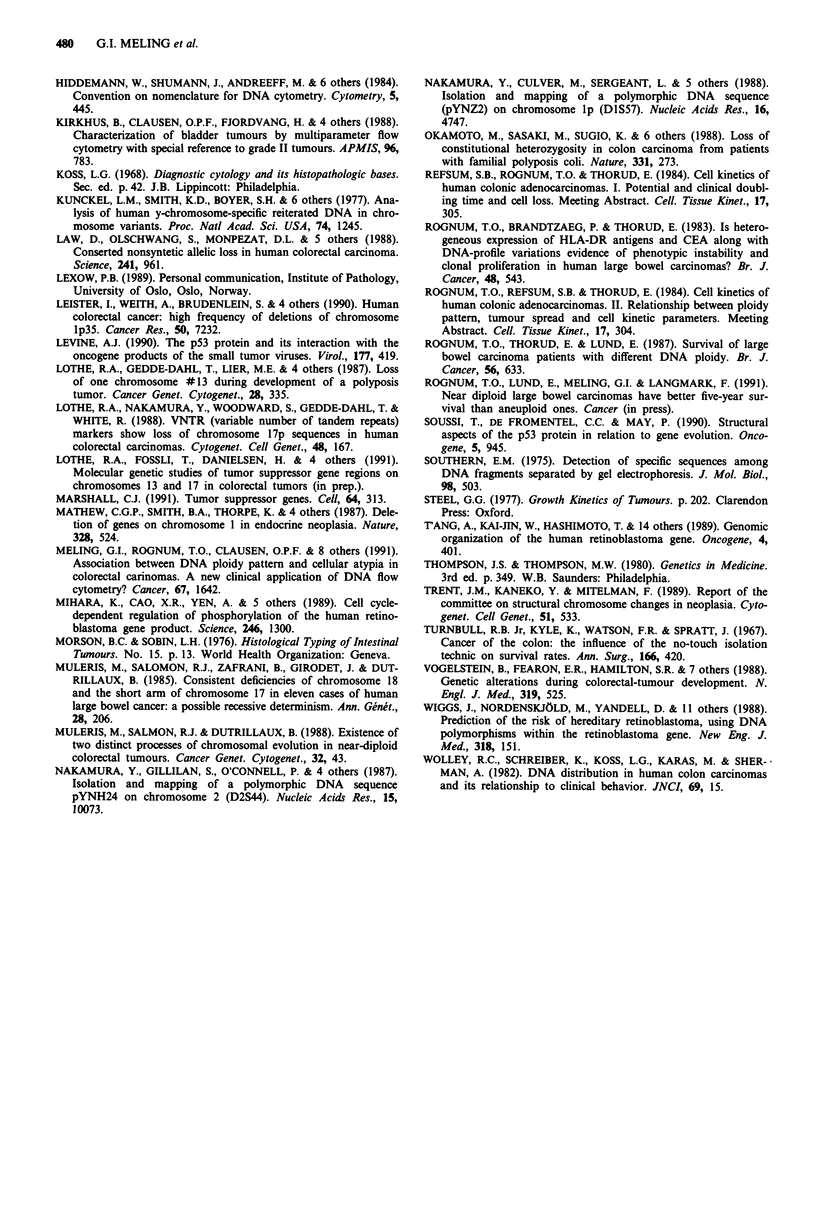# Genetic alterations within the retinoblastoma locus in colorectal carcinomas. Relation to DNA ploidy pattern studied by flow cytometric analysis.

**DOI:** 10.1038/bjc.1991.334

**Published:** 1991-09

**Authors:** G. I. Meling, R. A. Lothe, A. L. Børresen, S. Hauge, C. Graue, O. P. Clausen, T. O. Rognum

**Affiliations:** Institute of Forensic Medicine, National Hospital, University of Oslo, Norway.

## Abstract

**Images:**


Br. J. Cancer (1991), 64, 475–480 © Macmillan Press Ltd., 1991

### Genetic alterations within the retinoblastoma locus in colorectal carcinomas. Relation to DNA ploidy pattern studied by flow cytometric analysis

G.I. Meling[1], R.A. Lothe[3], A.-L. Børresen[3], S. Hauge[1], C. Graue[1], O.P.F. Clausen[2] & T.O. Rognum[1]

[1]*Institute of Forensic Medicine and* [2]*Institute of Pathology, The National Hospital, University of Oslo;* [3]*Department of Genetics, Institute for Cancer Research, The Norwegian Radium Hospital, Oslo, Norway.*

**Summary** Alterations within the retinoblastoma (Rb) gene, as detected by the VNTR probe p68RS2.0, and flow cytometric DNA pattern have been analysed in 255 colorectal carcinomas. A total of 35.3% of the tumours had alterations within the Rb gene. Amplification of one allele was demonstrated in 29.5% of the tumours, and loss of heterozygosity was found in 11.5%. No association was found between amplification within the Rb gene and clinicopathological characteristics of the patients. The high frequency of alterations demonstrated within the Rb gene, suggests that this gene is involved in colorectal carcinogenesis with amplification as by far the most abundant genetic alteration. This may imply that the Rb gene has an oncogene-like function in colorectal carcinomas, rather than acting as a tumour suppressor gene. Sixty-three per cent of the carcinomas were DNA aneuploid, and a significant association was demonstrated between amplification within the Rb gene and DNA aneuploidy ($P < 0.01$). Two other chromosome loci were analysed, on chromosome 1p (probe pYNZ2) and on chromosome 2p (probe pYNH24), respectively. On chromosome 1p, heterozygous loss was found in 22.2% of the tumours, indicating an involvement of this chromosome in a subset of colorectal carcinomas.

Recent studies have demonstrated that several genetic alterations may be involved in tumour development in the colorectum (for review, see Fearon & Vogelstein, 1990). A consistent observation is the loss or functional inactivation of several chromosome loci presumed to contain tumour suppressor genes. Loss of loci on chromosomes 5, 17 and 18, as well as mutations in the *ras* gene and the p53 gene have been reported (Muleris *et al.*, 1985; Fearon *et al.*, 1987; Vogelstein *et al.*, 1988; Lothe *et al.*, 1988; Okamoto *et al.*, 1988; Law *et al.*, 1988; Soussi *et al.*, 1990; Baker *et al.*, 1990), and it has been demonstrated that these changes often appear in a sequential manner, paralleling the clinical advancement of the tumours (Vogelstein *et al.*, 1988).

The retinoblastoma (Rb) gene is located on chromosome band 13q14.2. Loss or inactivation of this gene has been demonstrated in several human malignancies (for reviews, see Benedict *et al.*, 1990; Marshall, 1991). The Rb gene has been shown to suppress tumour development (Bookstein *et al.*, 1990), and the gene is therefore classified as a tumour suppressor gene. Little is known about the involvement of this gene in colorectal tumourigenesis. However, in a small series of colorectal carcinomas, it has recently been reported a significant amplification of the Rb gene (Gope *et al.*, 1990).

In DNA flow cytometric assays, the total DNA content of the main cell populations of tumours is quantified. Demonstration of an aneuploid DNA pattern reflects gross chromosomal changes, and may indicate genetic instability in the tumour cells (Rognum *et al.*, 1983; Meling *et al.*, 1991). Furthermore, the presence of aneuploidy is an important prognostic variable in colorectal carcinomas (Wolley *et al.*, 1982; Armitage *et al.*, 1985; Rognum *et al.*, 1987, Rognum *et al.*, 1991).

In 255 colorectal carcinomas, we have studied Rb gene alterations using Southern analysis, and DNA ploidy pattern using flow cytometric analysis, and examined the relationship between these two variables.

Correspondence: G.I. Meling, Institute of Forensic Medicine, The National Hospital, University of Oslo, 0027 Oslo 1, Norway.
Received 26 February 1991; and in revised form 18 April 1991.

### Materials and methods

### *Patients and tumour samples*

Fresh tissue samples from 255 colorectal adenocarcinomas removed during laparotomy from 129 men and 126 women were studied. Nine patients had more than one carcinoma synchronously. From these patients, only one tumour (chosen at random) was studied to ensure mutual independence of the tumours included. Clinicopathological characteristics of the patients are listed in Table I.

Single cell suspensions were prepared either immediately after tumour excision or after overnight storage in ice-cold phosphate-buffered saline (PBS), pH 7.6. The tumour samples were mechanically minced in PBS, followed by nylon mesh filtration (mesh pore size 70 µm) (Seidengazefabrik AG Thal, Switzerland). The cells were both fixed and stored in 70% ethanol at 4°C, until flow cytometric analysis or DNA extraction was performed.

To evaluate the contamination of normal cells in the tumour cell suspensions, cytospin preparations were made from ethanol fixed cell suspensions from 18 of the tumours. The cells were stained according to a modified Papanicolaou method (Lexow, 1989). A minimum of 300 cells from each tumour were evaluated and classified as either tumour cells, mononuclear leukocytes, granulocytes, or cells of undetermined origin, according to standard cytological criteria (Koss, 1968).

To evaluate intratumoural variation, two to five samples from each of eight carcinomas were analysed for DNA alterations.

### *Southern analysis*

Nuclear DNA was extracted from both tumour cell suspensions and from peripheral blood leukocytes in a 340A Nucleic Extractor (Applied Biosystem, Rotterdam, The Netherlands), using standard methods (phenol-chloroform extraction and ethanol precipitation) (Kunckel *et al.*, 1977). DNA samples (7.5 µg) were digested to completion with approximately eight times excess of the restriction enzymes RsaI, PvuII, and MspI (Amersham, Buckinghamshire, England), respectively. RsaI digested DNA was electrophoresed

**Table I** Clinicopathological characteristics of the 255 colorectal carcinoma patients

| *Clinicopathological characteristics* | | *No. of tumours (%)* |
|---|---|---|
| *Dukes' stage*[a] | A | 34 (13%) |
| | B | 110 (43.2%) |
| | C | 76 (29.8%) |
| | D | 35 (13.7%) |
| *Histological grade*[b] | | |
| Well differentiated | | 13 (5.1%) |
| Moderately differentiated | | 207 (81.2%) |
| Poorly differentiated | | 35 (13.7%) |
| *Degree of cellular atypia*[c] | | |
| Slight cellular atypia | | 8 (3.1%) |
| Moderate cellular atypia | | 172 (67.5%) |
| Severe cellular atypia | | 75 (29.4%) |
| *Localisation*[d] | | |
| Right colon | | 79 (31.0%) |
| Left colon | | 64 (25.1%) |
| Rectum | | 112 (43.9%) |

[a]According to the modified Dukes' classification (Dukes, 1932; Turnbull *et al.*, 1967); [b]According to the criteria of the WHO (Morson & Sobin, 1976); [c]According to standard cytological criteria (Koss, 1966); [d]Carcinomas in the colon located proximal and distal to the midtransverse colon, are classified right- and left-sided, respectively.

on 2.0% NuSieve agarose gels (Bio Products, Vallenback Strand, Denmark) for 60 h at 38 V. The digests of PvuII and MspI were separated in 1.0% agarose gels (Sigma Chemical Co., St. Louis, MO, USA) for 30 h at 48 V. The DNA was transferred onto Nylon membranes (Bio-Rad, Richmond, CA, USA) according to a slightly modified Southern procedure (Southern, 1975), using alkaline solution (0.4 M NaOH and 0.6 M NaCl). Southern blots of RsaI digested DNA were hybridised with the probe p68RS2.0 which detects a variable number of tandem repeat (VNTR) region in an intron of the Rb gene (Wiggs *et al.*, 1988). As a control for a possible background level of DNA alterations, the Southern blots of PvuII and MspI digested DNA were hybridised with the VNTR probes pYNZ2, which maps to a sequence on chromosome 1p, (Nakamura *et al.*, 1988) and pYNH24 which maps to a sequence on chromosome 2p (Nakamura *et al.*, 1987), respectively (Table II).

The probes were radioactively labelled with [$\alpha^{32}$P]dCTP according to the random labelling method (Feinberg & Vogelstein, 1983). Prehybridisations (2 h) and hybridisations (overnight) were carried out in 0.5 M $NaHPO_4$, pH 7.2, 0.001 M EDTA, 7% Sodium Dodecyl Sulfate (SDS), and 1% Bovine Serum Albumin (BSA) at 65°C. After hybridisation, the filters were washed for 10–15 min at 65°C in a 0.04 M $NaHPO_4$ solution, pH 7.2, containing 1% SDS. X-ray films (Kodak, XAR-5, Eastman Kodak Company, Rochester, NY, USA) were exposed to the radiolabelled filters for 1–7 days at −70°C using intensifying screens (Kodak).

*Densitometric measurements and scoring criteria*

Densitometric measurements were performed on all the heterozygote cases using a Bio-Rad 1650 scanning densitometer. The membranes were rehybridised with an additional probe to adjust for differences in DNA loading (Table II). When the ratio between the allele ratios of normal and tumour DNA was $\geqslant 1.5$, a significant change was scored. This ratio was chosen to be able to score a tumour with the change in question common for one-third or 50% (reduction or increase in hybridisation intensity, respectively) or more of the tumour cells (with regard to allelic amplification, assuming that the affected cells each had one extra copy of the DNA segment analysed). After adjustment for amount of DNA loaded, the genetic change was scored as either allelic amplification, when an increase in hybridisation intensity of one allele was observed, or loss of heterozygosity, when a reduction was observed (Figure 1).

**Table II** Restriction endonucleases and VNTR DNA probes used

| *Probe*[a] | *Chromosomal location* | *Restriction endonuclease* | *Detected level of heterozygosity* | *Probe used as correction for loading*[a] |
|---|---|---|---|---|
| p68RS2.0 | 13q14.2 | RsaI | 0.61 | pYNZ2 (D1S57) |
| pYNZ2 (D1S57) | 1p | PvuII | 0.71 | pTHH59 (D17S4) |
| pYNH24 (D2S44) | 2p | MspI | 0.93 | pRMU3 (D17S24) |

[a]In the brackets are given the D-numbers according to the nomenclature of the HGM10 (1989).

*Flow cytometric analysis*

Flow cytometric analysis was performed on the ethanol fixed cell suspensions according to the method described by Crissman and Steinkamp (1982), and modified by Kirkhus *et al.* (1988). The cells were incubated with RNAase, 190 $\mu$g ml$^{-1}$ (Boehring, Mannheim, Germany), for 30 min in dark in 20°C, and thereafter stained with the fluorochrome propidium iodide, 17 $\mu$g ml$^{-1}$ (Sigma Chemical Co., St Louis, MO, USA), for 1 h on ice in dark. The emission of red fluorescence was measured in an Ortho Cytofluorograph 50H (Ortho Instruments, Westwood, MA, USA). Mouse spleen lymphocytes were used as an external diploid (2c) DNA control.

*Definition of aneuploidy*

The cellular amount of DNA was expressed as a DNA index (Hiddemann *et al.*, 1984). The DNA index is the ratio between the mean relative DNA content of the cell population examined and the mean DNA content of the diploid reference cells. The peak with the lowest DNA content was referred to as the diploid reference cell population (Meling *et al.*, 1991). The mean coefficient of variation (CV) of the diploid peak was 3.2 (range 1.1–5.5). A tumour was defined as aneuploid (Figure 2b) if a second distinct population of $G_1$ cells was present, and had a DNA index $\geqslant 1.10$ (Kirkhus *et al.*, 1988; Meling *et al.*, 1991). Otherwise the tumour was defined as near diploid (Figure 2a). This definition is at variance with the definition of aneuploidy based on cytogenetic criteria, which defines tumours with any deviation from the exact multiple of the haploid chromosome number, as aneuploid (Thompson & Thompson, 1980).

In the DNA histogram analysis, minor peaks at the 4c level (Figure 2a) may represent either $G_2$ cells of the diploid $G_1$ population, or $G_1$ cells of a tetraploid (aneuploid) cell population, or a combination of the two. To differentiate between an aneuploid cell population with tetraploid DNA content and $G_2$ cells of the diploid population in the DNA histograms, planimetry was used (Göhde, 1973). When the area below the 4c peak was larger than the area between the 2c and 4c levels (S-phase), the 4c peak was considered to represent an aneuploid cell population. The rationale for this is that in proliferating mammalian cells, the proportion of cells in $G_2$ phase is generally lower than that in the S-phase (Steel, 1977).

*Statistical analysis*

For comparison between distributions, the $\chi^2$ test was applied.

### Results

*Proportions of tumour cells and non-tumour cells*

The cell suspensions contained a mean of 84% tumour cells (range 62 to 97%) with a normal distribution among the 18 different samples analysed. The non-tumour cells comprised a mean of 9% mononuclear leukocytes (range 3 to 24%), 1% granulocytes (range 0 to 4%), and 6% cells of undetermined origin (range 0 to 18%). The majority of the latter cells which had small and pycnotic nuclei, most likely represented tumour cells or small inflammatory cells.

**Figure 1** Allelic changes within the Rb locus detected by the VNTR probe p68RS2.0 on RsaI blots. DNA from normal (N) and tumour (T) tissue of four patients, all constitutionally heterozygote are shown. Rehybridisation with the probe pYNZ2 were performed to adjust for the amount of DNA loaded. Patient C1166 has no detectable change in the tumour. Patient C1088 has an amplification of one allele (2.0 fold increase in hybridisation intensity). Patient C1365 has an amplification of one allele (2.1 fold), and loss of the other allele. Patient C950 has lost one allele.

*Intratumoural homogeneity*

In seven of the eight carcinomas, no intratumoural variation was found in any of the loci analysed. In one carcinoma, heterozygous loss was found in the locus detected by probe pYNZ2 in four of five samples, while the findings in the other two loci were equal in all five samples.

*Southern analysis*

Of the 255 samples of tumour DNA and corresponding normal DNA tested, the proportions of heterozygote (informative) cases were as follows: 156 (61.2%) for the VNTR locus within the Rb gene, 180 (70.6%) for the VNTR locus detected by the probe pYNZ2, and 236 (92.5%) for the VNTR locus detected by the probe pYNH24 (Table II). The proportions of tumours with genetic changes are given as proportions of these informative cases.

**Figure 2a** Typical DNA histogram from one near diploid carcinoma. The histogram shows a single near diploid cell population (2c) and its corresponding $G_2$ fraction (4c). **b**, typical DNA histogram from one aneuploid carcinoma. The first peak represents a diploid cell population (2c), and the second an aneuploid cell population (AN) (DNA index = 1.6). The two smaller peaks to the right represent cell clumping and the $G_2$ fraction of the aneuploid cell population, respectively.

Amplification of one allele within the Rb locus was found in 46 of 156 cases (29.5%) (Figure 1 and Tables III and IV). The mean increase in hybridisation intensity of the amplified allele was 2.4-fold (range 1.5 to 4.2). Nine (5.8%) of the tumours with allelic amplification had lost the other allele. A total of 18 tumours (11.5%) had loss of one allele within the Rb locus (Figure 1 and Tables III and IV).

In the pYNZ2 locus on chromosome 1p, three of 180 carcinomas (1.7%) demonstrated allelic amplification, with increases in hybridisation intensity of 2.4, 3.8 and 4.0, respectively. One (0.6%) of these tumours had in addition loss of the other allele. A total of 40 tumours (22.2%) had heterozygous loss at this locus (Table III).

In the pYNH24 locus on 2p, six of 236 tumours (2.5%) had amplification of one allele, of which three (1.2%) had lost the other allele. The mean increase in hybridisation intensity was 3.0-fold (range 1.6 to 5.8). At this locus, 37 tumours (15.7%) had loss of heterozygosity (Table III).

The frequency of changes on chromosome 1p and 2p were similar in tumours with changes in the Rb locus ($n = 55$) (Table IV) and in those without changes in the Rb locus ($n = 101$, individual data not shown) ($\chi^2 = 0.7$, n.s. and $\chi^2 = 0.05$, n.s., respectively).

The frequency of allelic amplification was significantly higher within the Rb locus than on chromosomes 1p and 2p, respectively ($\chi^2 = 51.9$, $P < 0.0001$ and $\chi^2 = 59.3$, $P < 0.0001$). Similar frequencies of allelic loss were found within the Rb locus and at the locus on chromosome 2p ($\chi^2 = 1.3$, n.s.). There were significantly more tumours with heterozygous loss on chromosome 1p than within the Rb locus ($\chi^2 = 6.7$, $P < 0.01$), and there tended to be more tumours with heter-

**Table III** Frequencies of genetic alterations found in the 255 colorectal carcinomas

| Probe | Amplification | Heterozygous loss | Amplification and loss | Total |
|---|---|---|---|---|
| p68RS2.0 | 29.5% | 11.5% | 5.8% | 35.3% |
| pYNZ2 | 1.7% | 22.2% | 0.6% | 23.3% |
| pYHN24 | 2.5% | 15.7% | 1.2% | 16.9% |

ozygous loss on chromosome 1p than on chromosome 2p ($\chi^2 = 2.9$, $P<0.1$).

*DNA ploidy pattern and genetic alterations*

One hundred and sixty of the 255 carcinomas (62.7%) had aneuploid DNA pattern as demonstrated by DNA flow cytometric analysis (Figure 2).

A significant association was found between amplification within the Rb gene and DNA aneuploidy, as 36 of 46 tumours (78.3%) with allelic amplification were aneuploid, compared to 55 of 101 tumours (54.5%) without genetic changes in the Rb locus ($\chi^2 = 7.6$, $P<0.01$) (Table V).

Significantly more tumours with heterozygous loss on chromosomes 1p ($\chi^2 = 11.3$, $P<0.001$) and 2p ($\chi^2 = 9.0$, $P<0.01$) were aneuploid (85% and 83.8%, respectively) than those without alterations on these chromosomes (55.8% and 57.7%, respectively).

Of a total of 57 aneuploid tumours that were informative at all three loci, 23 (40.4%) had amplification within the Rb gene. Only one tumour (2.1%) showed allelic amplification on chromosome 1p, and another one (2.1%) showed allelic amplification on chromosome 2p.

*Genetic alterations and clinicopathological characteristics*

Similar distributions of Dukes' stage, histological grade, degree of cellular atypia, and tumour localisation were found in the tumours with amplification in the Rb locus ($n = 46$) compared with those without Rb gene changes ($n = 101$) (Table V).

### Discussion

Colorectal carcinomas are likely to develop as a result of progressive accumulation of several genetic alterations (for review, see Fearon & Vogelstein, 1990). The majority of genetic changes detected have involved mutational inactivation or loss of putative tumour suppressor genes. So far, however, only few cases of gene amplifications have been reported, involving only oncogenes. Consequently, amplifications have been assumed to play a minor role in colorectal carcinogenesis.

The retinoblastoma gene acts as a tumour suppressor gene, and is known to be involved in several human malignancies (for reviews, see Benedict *et al.*, 1990; Marshall, 1991). In a cytogenetic study on colorectal carcinomas, a non-random gain of chromosome 13 has been demonstrated (Muleris *et al.*, 1988), and in a smaller colorectal carcinoma study, a significant amplification of the Rb gene was demonstrated both at the DNA and RNA levels (Gope *et al.*, 1990), both studies suggesting that the Rb gene may be involved in colorectal carcinogenesis. The findings of the latter report have recently been confirmed by Lothe *et al.* (1991). Furthermore, loss of one chromosome 13 during development of a polyposis tumour has earlier been reported (Lothe *et al.*, 1987).

In our study of 255 colorectal carcinomas, we found genetic alterations within the Rb locus in 35.3% of the informative tumours. The majority of these tumours (46 of 55, 84%) had amplification of one allele, whereas only nine (16%) had heterozygous loss within the Rb locus without allelic amplification of the remaining allele. Our results therefore confirm that the Rb gene may be involved in colorectal carcinogenesis, and furthermore, that allelic amplification is the genetic change of importance.

**Table IV** DNA ploidy patterns and genetic alterations in the 55 colorectal carcinomas with changes within the Rb locus

| Patient code | DNA ploidy | Rb changes | Fold increase of one Rb allele | 1p changes | 2p changes |
|---|---|---|---|---|---|
| C 848 | AN | ● | 1.8 | + | + |
| C 887 | AN | ● | 2.1 | + | + |
| C 922 | AN | ● | 3.7 | – | ■ |
| C 936 | AN | ● | 2.0 | ● | + |
| C 937 | AN | ● | 1.5 | + | + |
| C 940 | AN | ● | 4.0 | + | + |
| C1008 | AN | ● | 4.2 | + | + |
| C1010 | AN | ● | 2.0 | – | + |
| C1013 | AN | ● | 1.6 | + | + |
| C1025 | AN | ● | 3.5 | + | + |
| C1041 | AN | ● | 2.0 | – | + |
| C1045 | AN | ● | 2.4 | – | + |
| C1048 | AN | ● | 2.6 | + | + |
| C1088 | AN | ● | 2.0 | – | + |
| C1089 | AN | ● | 1.8 | + | – |
| C1096 | AN | ● | 2.0 | + | + |
| C1102 | AN | ● | 2.9 | + | + |
| C1194 | AN | ● | 3.5 | – | – |
| C1195 | AN | ● | 3.5 | + | + |
| C1263 | AN | ● | 2.3 | – | ■ |
| C1284 | AN | ● | 3.9 | + | + |
| C1291 | AN | ● | 2.5 | + | + |
| C1302 | AN | ● | 4.0 | + | ■ |
| C1332 | AN | ● | 3.0 | + | + |
| C1334 | AN | ● | 2.0 | ■ | ● |
| C1340 | AN | ● | 2.0 | – | + |
| C1364 | AN | ● | 2.0 | – | – |
| C1382 | AN | ● | 2.5 | ■ | + |
| C1400 | AN | ● | 2.2 | ■ | + |
| C1403 | AN | ● | 1.9 | – | + |
| C1406 | AN | ● | 3.3 | ■ | + |
| C 850 | ND | ● | 1.9 | – | + |
| C 946 | ND | ● | 1.8 | – | + |
| C1099 | ND | ● | 1.5 | – | – |
| C1276 | ND | ● | 2.0 | ■ | ■ |
| C1324 | ND | ● | 2.0 | – | + |
| C1343 | ND | ● | 2.5 | + | – |
| C 904 | AN | ▲ | 2.3 | – | + |
| C1014 | AN | ▲ | 2.5 | ■ | + |
| C1120 | AN | ▲ | 2.0 | + | ■ |
| C1168 | AN | ▲ | 4.0 | – | + |
| C1365 | AN | ▲ | 2.1 | + | – |
| C 945 | ND | ▲ | 2.0 | – | + |
| C 982 | ND | ▲ | 2.0 | + | + |
| C1024 | ND | ▲ | 2.3 | ■ | + |
| C1103 | ND | ▲ | 2.1 | – | + |
| C 914 | AN | ■ | | – | + |
| C1155 | AN | ■ | | – | ■ |
| C1290 | AN | ■ | | ■ | + |
| C1447 | AN | ■ | | ■ | + |
| C 858 | ND | ■ | | + | + |
| C 950 | ND | ■ | | – | – |
| C 965 | ND | ■ | | – | + |
| C1030 | ND | ■ | | – | + |
| C1389 | ND | ■ | | – | + |

Information about the carcinomas are given, where a genetic alteration was detected with the probe p68RS2.0, homologous to a VNTR region within the Rb gene; AN: aneuploid DNA pattern; ND: near diploid DNA pattern; ●: Heterozygote with allelic amplification of one allele; ▲: Loss of heterozygosity and amplification of the remaining allele; ■: Loss of heterozygosity; –: Homozygote; +: Heterozygote without changes.

In other malignancies with assumed involvement of the Rb gene in the tumourigenesis, loss of heterozygosity has been demonstrated, indicating a tumour suppressor gene activity of the Rb gene (Hansen & Cavenee, 1987). Since heterozygous loss within the Rb gene does not occur more frequently than in random genes of chromosome 1 or 2, there seems to be no role for the Rb gene as a tumour suppressor gene in colorectal carcinomas. In the present study, however, we found that allelic amplification was by far the most common change within the Rb gene. Since amplification is a

**Table V** Clinicopathological characteristics of the patients according to amplification within the Rb gene

| *Clinicopathological characteristics* | *No. of cases with amplification within the RB gene n = 46 (%)* | *without Rb gene changes n = 101 (%)* | *Level of sign.* |
|---|---|---|---|
| *DNA ploidy pattern* | | | |
| Aneuploid | 36 (78) | 55 (54) | $P<0.01$ |
| Near diploid | 10 (22) | 46 (46) | |
| *Dukes' stage* A | 3 (6) | 14 (14) | |
| B | 23 (50) | 38 (37) | |
| C | 11 (24) | 34 (34) | n.s. |
| D | 9 (20) | 15 (15) | |
| *Histological grade* | | | |
| Well differentiated | 1 (2) | 4 (4) | |
| Moderately diff. | 44 (96) | 84 (83) | n.s. |
| Poorly diff. | 1 (2) | 13 (13) | |
| *Degree of cellular atypia* | | | |
| Slight | 3 (6) | 3 (3) | n.s. |
| Moderate | 26 (57) | 74 (73) | |
| Severe | 17 (37) | 24 (24) | |
| *Localisation* | | | |
| Right colon | 8 (17) | 31 (31) | n.s. |
| Left colon | 14 (31) | 23 (23) | |
| Rectum | 24 (52) | 47 (46) | |

phenomenon known to be associated with some oncogenes, our results suggest that the Rb gene may have an oncogene-like effect in colorectal carcinomas. Another gene involved in colorectal tumourigenesis, the p53 gene located on chromosome 17, has previously been shown to be able to act both as a tumour suppressor gene as well as, in a mutated form, an oncogene (for review, see Levine, 1990). Our demonstration of an increased copy number of a VNTR region within the Rb gene suggests that this region or the whole gene may be involved in local rearrangements, or that the carcinoma cells may contain increased copy numbers of large portions of chromsome 13. Preliminary data from our laboratory, however, indicate that amplification within the Rb locus does not commonly involve DNA sequences flanking the gene (data not shown), but indeed involves other DNA sequences within the gene itself (Lothe *et al.*, 1991). This makes it unlikely that the Rb gene is merely coamplified together with another possible oncogene located on chromosome 13. It has also recently been demonstrated that the DNA sequence detected by the VNTR probe p68RS2.0 contains possible hot spots for structural aberrations (T'Ang *et al.*, 1989).

We have earlier reported a higher proportion of cells in the S-phase of the cell cycle in aneuploid carcinomas compared with near diploid ones (Refsum *et al.*, 1984; Rognum *et al.*, 1984). In this study, we could demonstrate a significant association between amplification within the Rb gene and DNA aneuploidy. It has been assumed that both the Rb gene protein and p53 negatively regulate the passage of cells through the cell cycle (Mihara *et al.*, 1989; Diller *et al.*, 1990). In aneuploid carcinomas, an amplified Rb gene may have an adverse effect, and enhance the number of cells that go through the S-phase, either directly, or indirectly through an interaction with and inactivation of p53.

Among the 57 aneuploid tumours informative at all three loci analysed, we could demonstrate only two carcinomas with DNA amplification on chromosomes 1p and 2p, respectively. This suggest that the observed gain in total DNA content in aneuploid cells is not merely the result of random amplification of the diploid genome.

The frequency of heterozygous loss was higher on chromosome 1p than on chromosome 2p and within the Rb gene. This is in correspondence with the recently reported loss on chromosome 1p in 42% of colon carcinomas (Leister *et al.*, 1990). Also, in several other solid tumours, heterozygous loss or cytogenetic deletions on chromosome 1p has been associated with tumour development (Brodeur *et al.*, 1981; Mathew *et al.*, 1987; Trent *et al.*, 1989). In the present study, allelic loss on chromosome 1 was significantly associated with DNA aneuploidy. Therefore, loss of DNA sequences on chromosome 1 may be of importance for aneuploidy in a subset of aneuploid colorectal carcinomas.

In conclusion, our results suggest that the Rb gene is involved in colorectal carcinogenesis. As allelic amplification was found to be the most frequent change within the Rb locus, the mechanism for involvement of this gene is probably different in colorectal carcinomas compared with retinoblastomas. Our results also indicate that the increased amount of DNA present in aneuploid cell populations may constitute specific gene amplifications of importance in the colorectal carcinogenesis. Follow-up studies are already in progress to evaluate the possible prognostic significance of amplifications within the Rb gene in colorectal carcinomas.

The authors are grateful to Drs: J.N. Wiig, O.C. Lunde, E. Schlichting, E. Trondsen, J. Hognestad, Ø. Havig and A. Bergan for supplementing the tumour samples used in the study, and to Dr T. Dryja for the p68RS2.0 probe, and Drs Y. Nakamura and R. White for the probes pYNZ2 and pYNH24. The authors thank Ms Y. Chen, Ms Aa. Schjølberg, and Ms B. Ringerike for technical assistance. The study is supported by the Norwegian Cancer Society, A/S Freia Chocolate Factory's Medical Fund, The Medical Innovation Foundation, and the Legacy of Astrid and Birger Thorsted, Oslo, Norway.

### References

ARMITAGE, N.C., ROBINS, R.A., EVANS, D.F., TURNER, D.R., BALDWIN, R.W. & HARDCASTLE, J.D. (1985). The influence of tumour cell DNA abnormalities on survival in colorectal cancer. *Br. J. Surg.*, **72**, 828.

BAKER, S.J., PREISINGER, A.C., JESSUP, J.M. & 5 others (1990). p53 gene mutations occur in combination with 17p allelic deletions as late events in colorectal tumorigenesis. *Cancer Res.*, **50**, 7717.

BENEDICT, W.F.,. XU, H.-J., HU, S.-X. & TAKAHASHI, R. (1990). Role of retinoblastoma gene in the initiation and progression of human cancer. *J. Clin. Invest.*, **85**, 988.

BOOKSTEIN, R., SHEW, J.-Y., CHEN, P.-L., SCULLY, P. & LEE, W.-H. (1990). Suppression of tumorigenicity of human prostate carcinoma cells by replacing a mutated RB gene. *Science*, **247**, 712.

BRODEUR, G.M., GREEN, A.A., HAYES, F.A., WILLIAMS, K.J., WILLIAMS, D.L. & TSIATIS, A.A. (1981). Cytogenetic features of human neuroblastomas and cell lines. *Cancer Res.*, **41**, 4678.

CRISSMAN, H.A. & STEINKAMP, J.A. (1982). Rapid, one step staining procedures for analysis of cellular DNA and protein by single and dual laser flow cytometry. *Cytometry*, **3**, 84.

DILLER, L., KASSEL, J., NELSON, C.E. & 7 others (1990). p53 functions as a cell cycle control protein in osterosarcomas. *Mol. Cell Biol.*, **10**, 5772.

DUKES, C.E. (1932). The classification of cancer of the rectum. *J. Pathol. Bacteriol.*, **35**, 323.

FEARON, E.R., HAMILTON, S.R. & VOGELSTEIN, B. (1987). Clonal analysis of human colorectal tumors. *Science*, **238**, 193.

FEARON, E.R. & VOGELSTEIN, B. (1990). A genetic model for colorectal tumorigenesis. *Cell*, **61**, 759.

FEINBERG, A.P. & VOGELSTEIN, B. (1983). A technique for radiolabeling DNA restriction endonuclease fragments to high specific activity. *Analyt. Biochem.*, **132**, 6.

GOPE, R., CHRISTENSEN, M.A., THORSON, A. & 6 others (1990). Increased expression of the retionoblastoma gene in human colorectal carcinomas relative to normal colonic mucosa. *JNCI*, **82**, 310.

GÖHDE, W. (1973). Zellzyclusanalysen mit dem Impulscytophotometer: der einfluss chemischer noxen auf prolieferationskinetik von tumor zellen. p. 429. Thesis: Münster.

HANSEN, M.F. & CAVENEE, W.K. (1987). Genetics of cancer predisposition. *Cancer Res.*, **47**, 5518.

HGM10. TENTH INTERNATIONAL WORKSHOP ON HUMAN GENE MAPPING (1989). *Cytogenet. Cell Genet.*, **51**, 622.

HIDDEMANN, W., SHUMANN, J., ANDREEFF, M. & 6 others (1984). Convention on nomenclature for DNA cytometry. *Cytometry*, **5**, 445.

KIRKHUS, B., CLAUSEN, O.P.F., FJORDVANG, H. & 4 others (1988). Characterization of bladder tumours by multiparameter flow cytometry with special reference to grade II tumours. *APMIS*, **96**, 783.

KOSS, L.G. (1968). *Diagnostic cytology and its histopathologic bases*. Sec. ed. p. 42. J.B. Lippincott: Philadelphia.

KUNCKEL, L.M., SMITH, K.D., BOYER, S.H. & 6 others (1977). Analysis of human y-chromosome-specific reiterated DNA in chromosome variants. *Proc. Natl Acad. Sci. USA*, **74**, 1245.

LAW, D., OLSCHWANG, S., MONPEZAT, D.L. & 5 others (1988). Conserted nonsyntetic allelic loss in human colorectal carcinoma. *Science*, **241**, 961.

LEXOW, P.B. (1989). Personal communication, Institute of Pathology, University of Oslo, Oslo, Norway.

LEISTER, I., WEITH, A., BRUDENLEIN, S. & 4 others (1990). Human colorectal cancer: high frequency of deletions of chromosome 1p35. *Cancer Res.*, **50**, 7232.

LEVINE, A.J. (1990). The p53 protein and its interaction with the oncogene products of the small tumor viruses. *Virol.*, **177**, 419.

LOTHE, R.A., GEDDE-DAHL, T., LIER, M.E. & 4 others (1987). Loss of one chromosome #13 during development of a polyposis tumor. *Cancer Genet. Cytogenet.*, **28**, 335.

LOTHE, R.A., NAKAMURA, Y., WOODWARD, S., GEDDE-DAHL, T. & WHITE, R. (1988). VNTR (variable number of tandem repeats) markers show loss of chromosome 17p sequences in human colorectal carcinomas. *Cytogenet. Cell Genet.*, **48**, 167.

LOTHE, R.A., FOSSLI, T., DANIELSEN, H. & 4 others (1991). Molecular genetic studies of tumor suppressor gene regions on chromosomes 13 and 17 in colorectal tumors (in prep.).

MARSHALL, C.J. (1991). Tumor suppressor genes. *Cell*, **64**, 313.

MATHEW, C.G.P., SMITH, B.A., THORPE, K. & 4 others (1987). Deletion of genes on chromosome 1 in endocrine neoplasia. *Nature*, **328**, 524.

MELING, G.I., ROGNUM, T.O., CLAUSEN, O.P.F. & 8 others (1991). Association between DNA ploidy pattern and cellular atypia in colorectal carinomas. A new clinical application of DNA flow cytometry? *Cancer*, **67**, 1642.

MIHARA, K., CAO, X.R., YEN, A. & 5 others (1989). Cell cycle-dependent regulation of phosphorylation of the human retinoblastoma gene product. *Science*, **246**, 1300.

MORSON, B.C. & SOBIN, L.H. (1976). *Histological Typing of Intestinal Tumours*. No. 15. p. 13. World Health Organization: Geneva.

MULERIS, M., SALOMON, R.J., ZAFRANI, B., GIRODET, J. & DUTRILLAUX, B. (1985). Consistent deficiencies of chromosome 18 and the short arm of chromosome 17 in eleven cases of human large bowel cancer: a possible recessive determinism. *Ann. Génét.*, **28**, 206.

MULERIS, M., SALMON, R.J. & DUTRILLAUX, B. (1988). Existence of two distinct processes of chromosomal evolution in near-diploid colorectal tumours. *Cancer Genet. Cytogenet.*, **32**, 43.

NAKAMURA, Y., GILLILAN, S., O'CONNELL, P. & 4 others (1987). Isolation and mapping of a polymorphic DNA sequence pYNH24 on chromosome 2 (D2S44). *Nucleic Acids Res.*, **15**, 10073.

NAKAMURA, Y., CULVER, M., SERGEANT, L. & 5 others (1988). Isolation and mapping of a polymorphic DNA sequence (pYNZ2) on chromosome 1p (D1S57). *Nucleic Acids Res.*, **16**, 4747.

OKAMOTO, M., SASAKI, M., SUGIO, K. & 6 others (1988). Loss of constitutional heterozygosity in colon carcinoma from patients with familial polyposis coli. *Nature*, **331**, 273.

REFSUM, S.B., ROGNUM, T.O. & THORUD, E. (1984). Cell kinetics of human colonic adenocarcinomas. I. Potential and clinical doubling time and cell loss. Meeting Abstract. *Cell. Tissue Kinet.*, **17**, 305.

ROGNUM, T.O., BRANDTZAEG, P. & THORUD, E. (1983). Is heterogeneous expression of HLA-DR antigens and CEA along with DNA-profile variations evidence of phenotypic instability and clonal proliferation in human large bowel carcinomas? *Br. J. Cancer*, **48**, 543.

ROGNUM, T.O., REFSUM, S.B. & THORUD, E. (1984). Cell kinetics of human colonic adenocarcinomas. II. Relationship between ploidy pattern, tumour spread and cell kinetic parameters. Meeting Abstract. *Cell. Tissue Kinet.*, **17**, 304.

ROGNUM, T.O., THORUD, E. & LUND, E. (1987). Survival of large bowel carcinoma patients with different DNA ploidy. *Br. J. Cancer*, **56**, 633.

ROGNUM, T.O., LUND, E., MELING, G.I. & LANGMARK, F. (1991). Near diploid large bowel carcinomas have better five-year survival than aneuploid ones. *Cancer* (in press).

SOUSSI, T., DE FROMENTEL, C.C. & MAY, P. (1990). Structural aspects of the p53 protein in relation to gene evolution. *Oncogene*, **5**, 945.

SOUTHERN, E.M. (1975). Detection of specific sequences among DNA fragments separated by gel electrophoresis. *J. Mol. Biol.*, **98**, 503.

STEEL, G.G. (1977). *Growth Kinetics of Tumours*. p. 202. Clarendon Press: Oxford.

T'ANG, A., KAI-JIN, W., HASHIMOTO, T. & 14 others (1989). Genomic organization of the human retinoblastoma gene. *Oncogene*, **4**, 401.

THOMPSON, J.S. & THOMPSON, M.W. (1980). *Genetics in Medicine*. 3rd ed. p. 349. W.B. Saunders: Philadelphia.

TRENT, J.M., KANEKO, Y. & MITELMAN, F. (1989). Report of the committee on structural chromosome changes in neoplasia. *Cytogenet. Cell Genet.*, **51**, 533.

TURNBULL, R.B. Jr, KYLE, K., WATSON, F.R. & SPRATT, J. (1967). Cancer of the colon: the influence of the no-touch isolation technic on survival rates. *Ann. Surg.*, **166**, 420.

VOGELSTEIN, B., FEARON, E.R., HAMILTON, S.R. & 7 others (1988). Genetic alterations during colorectal-tumour development. *N. Engl. J. Med.*, **319**, 525.

WIGGS, J., NORDENSKJÖLD, M., YANDELL, D. & 11 others (1988). Prediction of the risk of hereditary retinoblastoma, using DNA polymorphisms within the retinoblastoma gene. *New Eng. J. Med.*, **318**, 151.

WOLLEY, R.C., SCHREIBER, K., KOSS, L.G., KARAS, M. & SHERMAN, A. (1982). DNA distribution in human colon carcinomas and its relationship to clinical behavior. *JNCI*, **69**, 15.